# Salidroside regulates the expressions of IL-6 and defensins in LPS-activated intestinal epithelial cells through NF-κB/MAPK and STAT3 pathways

**DOI:** 10.22038/ijbms.2018.26994.6602

**Published:** 2019-01

**Authors:** Jiawen Wang, Yibin Pan, Yongqing Cao, Wei Zhou, Jingen Lu

**Affiliations:** 1 Department of Anal & Intestinal Disease, Longhua Hospital, Shanghai University of Traditional Chinese Medicine, Shanghai 200032, P.R.China

**Keywords:** Defensin, IL-6, Intestinal epithelial cell, MAPK, NF-κB, Salidroside, STAT3

## Abstract

**Objective(s):**

To reveal the detailed mechanism underlying the functions of salidroside on the inflammation of intestinal epithelial cells during IBD.

**Materials and Methods:**

Quantitative real-time PCR was employed to assess the expression of IL-6, IL-10, and α-defensins 5 and 6. ELISA assay was performed to measure the secretion of IL-6 and IL-10. MTT assay was used to determine the cell viability and proliferation. Western blot was used to assess the phosphorylation of NF-kB, Erk1/2, JNK, P38, JAK2, and STAT3.

**Results:**

Salidroside impaired the proliferation of intestinal epithelial cells at high concentrations (*P*< 0.05) and down-regulated interleukin-6 (IL-6) production induced by LPS (*P*<0.05). Western blot results showed that salidroside repressed the phosphorylation of NF-kB, Erk1/2, JNK, P38, JAK2 and STAT3 (*P*<0.05) and attenuated the activation of NF-κB, MAPK, and STAT3 pathways. Moreover, the expressions of α-defensin 5 and 6 were rescued by salidroside after LPS or SAC triggering (*P*<0.05).

**Conclusion:**

In summary, salidroside suppressed the expression of IL-6 and elevated the expression of defensins in LPS-activated intestinal epithelial cells through NF-κB/MAPK and STAT3 pathways. The mechanism revealed here may be potentially useful for the treatment of IBD with salidroside.

## Introduction

Enteritis is small intestine inflammation, which is often caused by eating or drinking things that are contaminated with bacteria or viruses. Besides germs, other factors, such as certain drugs, damages from radiation therapy as well as autoimmune conditions, could also result in enteritis. Clinicaly, there are mainly two types of chronic inflammatory bowel disease (IBD): Crohn’s disease (CD) and ulcerative colitis (UC). CD can affect any part of the gastrointestinal tract while UC is restricted to the colon and the rectum (1). The symptoms of common enteritis occur varying from hours to days after infection, which may include abdominal pain, acute and severe diarrhea, appetite loss, vomiting, and even rectal bleeding, severe internal cramps, and weight loss. People who get CD and UC may receive different treatments depending on their symptoms. The adverse effects and the cancer-inducing risk of drugs for IBD still restrain the clinical output of IBD treatment (2). So, developing new drugs, especially from Chinese traditional medicines, is an appealing and promising way to improve this situation.

Salidroside (SAL), with the chemical name p-hydroxyphenethyl-β-D-glucoside, is a biologically active component isolated from *Sargentodoxa cuneate* and *Rhodiola rosea *(3), which has various pharmacological properties, including anti-oxidant and anti-inflammatory (4, 5) effects as well as hepatoprotective (6), neuroprotective (7), and anti-cancer (8) functions. In previous reports, people found that salidroside could attenuate the inflammatory response in an ovalbumin (OVA)-induced asthma mouse model (9). Guan *et* *al.,* reported that salidroside could suppress the pro-inflammatory cytokine production and improve the survival in LPS-induced endotoxemia mouse model (10). Wang *et al.,* found that salidroside could repress the inflammation and adipogenesis in white adipose tissues and improve glucose homeostasis in obese mice (11). Taken together, salidroside has been confirmed to be a potent anti-inflammatory medicine and possess broad functions on different inflammatory diseases. However, the study about functions of salidroside on IBD is still lacking.

**Figure 1:**
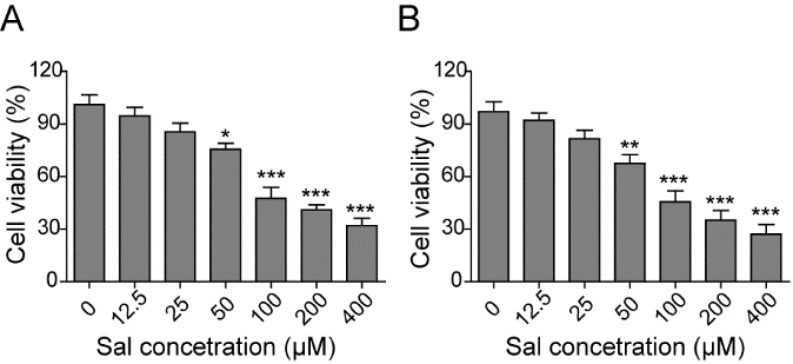
Salidroside showed cytotoxicity on intestinal epithelial cells

Rat intestinal epithelial cell line IEC-6 was treated without or with varying concentrations of salidroside for 24 hr (A) or 48 hr (B), and then the cell viability was measured with MTT kit. n=5. Error bars represented mean ± SD. *P*-values were determined by one-way analysis of variance (ANOVA) followed by Tukey’s *post hoc* test. *** *P*<0.001, ** *P*<0.01, and * *P*<0.05 vs control group. The result was a representative of three independent experiments

**Figure 2:**
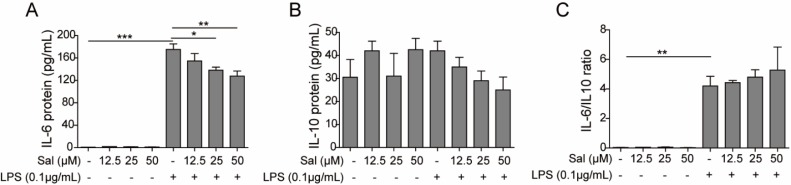
Salidroside suppressed the secretion of IL-6, not IL-10, in intestinal epithelial cells induced by LPS

Rat intestinal epithelial cell line IEC-6 was treated without or with low (12.5 µM), medium (25 µM), and high (50 µM) concentrations of salidroside for 2 hr and then further treated with 0.1 µg/ml LPS to induce the production of IL-6 (A) and IL-10 (B), which was measured by ELISA, control groups were only treated with the vehicle. (C) The ratio of IL-6 / IL-10 in A and B. n=3. Error bars represented mean ± SD. P-values were determined by one-way analysis of variance (ANOVA) followed by Tukey’s *post hoc* test. *** *P*<0.001, ** *P*<0.01, and * *P*<0.05 vs control group. The result was a representative of three independent experiments

**Figure 3:**
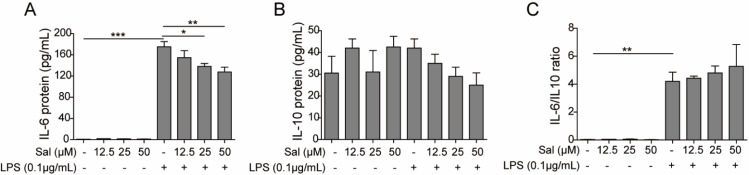
Salidroside attenuated the transcription of IL-6 in intestinal epithelial cells induced by LPS

Rat intestinal epithelial cell line IEC-6 was treated without or with low (12.5 µM), medium (25 µM), and high (50 µM) concentrations of salidroside for 2 hr, and then further treated with 0.1 µg/ml LPS to induce the production of IL-6 (A) and IL-10 (B), which was measured on transcriptional level by quantitative real-time PCR, control groups were only treated with the vehicle. (C) The ratio of IL-6 mRNA / IL-10 mRNA in A and B. n= 3. Error bars represented mean±SD. *P*-values were determined by one-way analysis of variance (ANOVA) followed by Tukey’s *post hoc* test. *** *P*<0.001, ** *P*<0.01, and * *P*<0.05 vs control group. The result was a representative of three independent experiments

**Figure 4:**
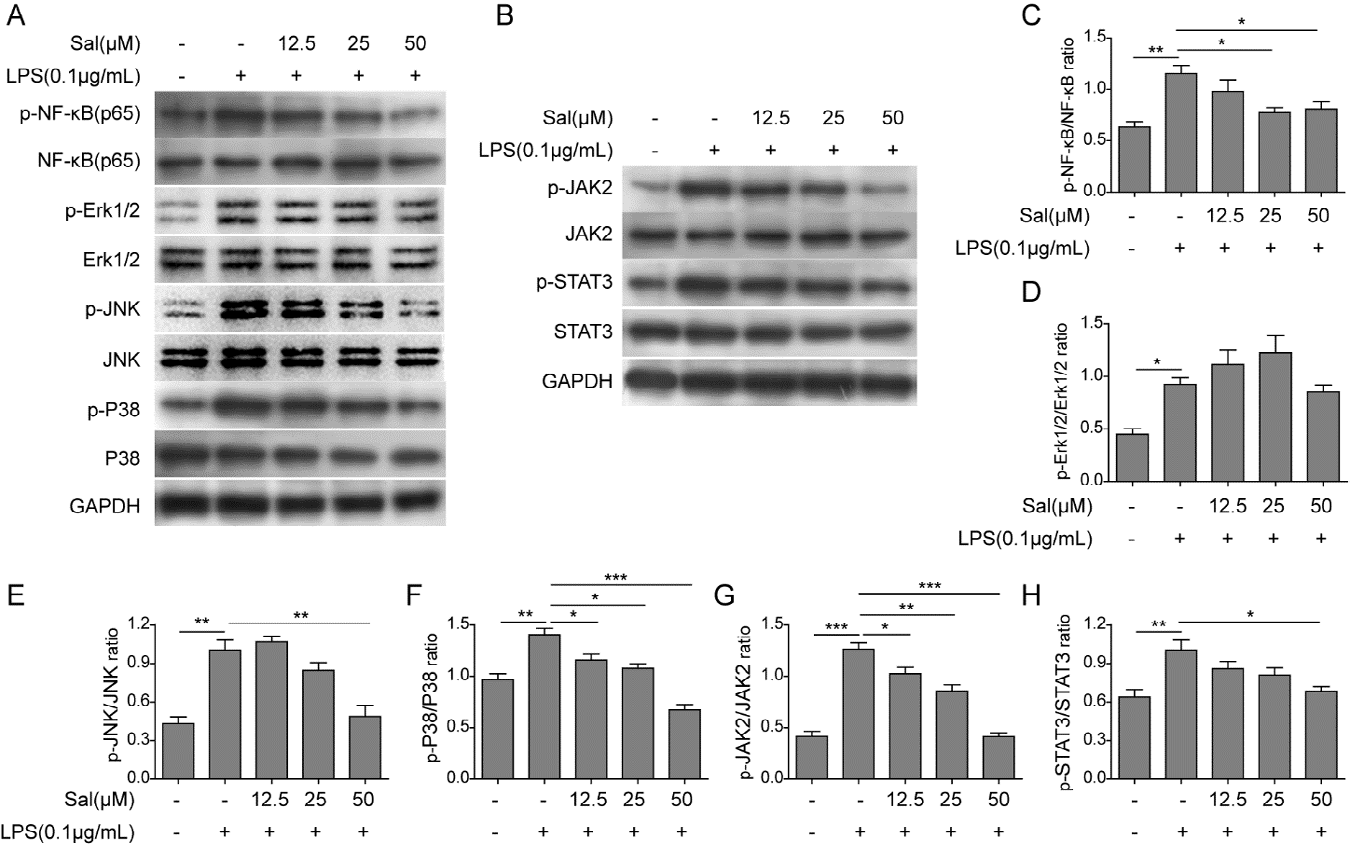
Salidroside suppressed the activation of NF-κB, MAPK, and STAT3 signaling pathways in intestinal epithelial cells induced by LPS

Rat intestinal epithelial cell line IEC-6 was treated without or with low (12.5 µM), medium (25 µM), and high (50 µM) concentrations of salidroside for 2 hr, and then further treated with 0.1 µg/ml LPS to induce the activation of NF-κB, MAPK, and STAT3 signaling pathways, which were indicated by the increased phosphorylation levels of representative proteins that were measured by Western blot (A-B), control groups were only treated with the vehicle. The phosphorylation levels of NF-kB (C), Erk1/2 (D), JNK (E), P38 (F), JAK2 (G), and STAT3 (H) were normalized to their inputs, respectively. n=3. Error bars represented mean±SD. P-values were determined by one-way analysis of variance (ANOVA) followed by Tukey’s *post hoc* test. *** *P*<0.001, ** *P*<0.01, and * *P*<0.05 vs control group. ns, not significant. The result was a representative of three independent experiments

**Figure 5:**
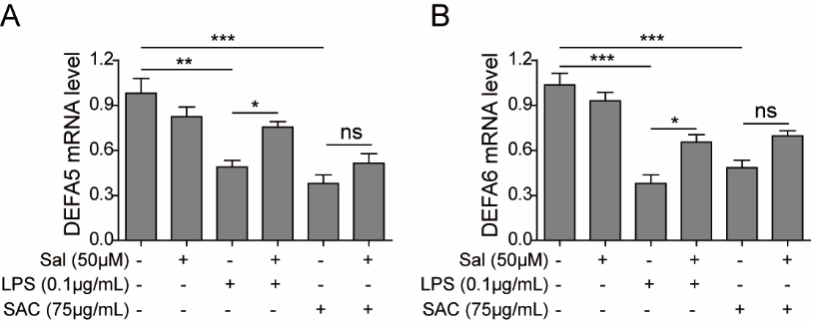
Salidroside restored the expressions of HD-5 and HD-6 in intestinal epithelial cells, which were down-regulated by LPS or SAC triggering

Rat intestinal epithelial cell line IEC-6 was treated without or with high (50 µM) concentrations of salidroside for 2 hr, and then further treated with 0.1 µg/ml LPS or 75 µg/ml SAC. The transcriptional levels of HD-5 (A) and HD-6 (B) were measured by quantitative real-time PCR, control groups were only treated with the vehicle. n=3. Error bars represented mean±SD. P-values were determined by one-way analysis of variance (ANOVA) followed by Tukey’s *post hoc* test. *** *P*<0.001, ** *P*<0.01, and * *P*<0.05 vs control group. ns, not significant. The result was a representative of three independent experiments

RIn recent years, people have been aware that the intestinal epithelium, the microbiota, and the host immune system cooperate to maintain intestinal homeostasis (12). Among the finely-tuned complex interaction networks, intestinal epithelium serves as a physical barrier which can segregate the commensal bacteria and absorb helpful nutrition. Moreover, enterocytes of the intestinal epithelium express toll-like receptors, nucleotide oligomerization domain (NOD)-like receptors, and RIG-I-like receptors that can recognize various pathogens and contribute to immune surveillance (13), so they are critical mediators of intestinal homeostasis. In addition, various cytokines and their receptors in the immune system also play crucial roles in the maintenance of intestinal homeostasis. Previous work clearly demonstrated that IL-6 could promote intestinal inflammation and cancer development (14), whereas interleukin-10 (IL-10) coordinated regulatory T cells to suppress inflammation and carcinogenesis (15). Moreover, intestinal epithelial cells produce and secrete a variety of antimicrobial peptides, e.g., defensins, into the mucosa and lumen that can contribute to barrier function in human gut (16). Due to the essential functions of IL-6, IL-10, and defensins in intestinal homeostasis, the regulation of these three factors deserves careful investigation.

As for the induction of inflammation and the production of pro-inflammatory cytokines, previous studies showed that several canonical signaling pathways were involved in this process, such as the Nuclear factor-kappa B (NF-kB) pathway, Mitogen-activated protein kinases (MAPKs) pathway, and Janus kinase-signal transducers and activators of transcription (JAK-STATs) pathway. NF-kB, which is a ubiquitous transcription factor that promotes gene expression of pro-inflammatory cytokines and chemokines, plays a key role in inflammatory and immune responses (17). Furthermore, extensive studies have found that activated NF-κB proteins have been related to inflammatory bowel disease (18) and other autoimmune diseases (19, 20). It is reported that an IκB kinase inhibitor, BMS-345541, could significantly reduce the severity of dextran sulfate sodium-induced colitis in mice (21). However, NF-κB also had anti-inflammatory functionh; it wasdproven that intestinal epithelial-cell-specific IKKγ (NEMO) deficient mice showed severe and spontaneous chronic intestinal inflammation (22). MAPKs, which consist of several serine and threonine kinases, also have critical functions in the induction of inflammation. These kinases can be classified into three major groups: p38, extracellular signal-related kinase 1/2 (Erk 1/2), and c-Jun N-terminal kinase 1/2 (JNK 1/2) (23). Among them, p38 and JNK MAPKs show increased phosphorylation level in the inflamed tissue from IBD patients (24, 25), and some clinical studies have been carried out to cure IBD or other autoimmune diseases with small molecule inhibitors of MAKs.. JAK-STATs is another important signaling pathway activated by various extracellular signaling ligands (26). The ligand engagement can result in the phosphorylations of JAKs and its downstream transcription factors STATs. Phosphorylated STATs then translocate into the nucleus to regulate the expression of inflammation-related genes ^23^. This pathway also contributes to the pathogenesis of some inflammatory and autoimmune diseases, including rheumatoid arthritis, psoriasis, and IBD (27). Among STAT families, STAT3 is the direct downstream of IL-6 and is a critical factor of Th17-mediated autoimmune processes. Previous genome-wide association studies (GWAS) howedshown that STAT3 gene has a crucial role in IBD susceptibility (28), and gain-of-function mutations in STAT3 cause autoimmune cytopenias and multiorgan autoimmunity (lutinegastrointestinal, and liver) in patients (29), which make STAT3 an appealing therapeutic target for such autoimmune diseases.

However, until nowble study has been carried out to assess the effects of salidroside treatment on enteritis and IBD. In this study, we utilized LPS to treat intestinal epithelial cells to obtain an IBD model *in vtro*, and then explored the functions of salidroside on treating IBD as well as the underlying mechanisms. 

## Materials and Methods

***Reagents***

Salidroside, Lipopolysaccharide (LPS), *Staphylococcus aureus* strain Cowan (SAC), DMSO and isopropanol were obtained from Sigma-Aldrich. Dulbecco’s modified Eagle’s medium (DMEM), fetal bovine serum (FBS), penicillin/streptomycin, bovine insulin, L-glutamine, 0.25% trypsin-EDTA, TRIzol, and MTT kit were obtained from Thermo Fisher Scientific. The first strand ceticsynthesis kit was from TaKaRa. SYBR® Green Master Mix was from Bio-Rad. Anti-NF-κB, anti-p-NF-κB, anti-Erk1/2, anti-p-Erk1/2, anti-JNK, anti-p-JNK, anti-P38, anti-p-P38, anti-JAK2, anti-p-JAK2, anti-STAT3, anti-p-STAT3, and anti-GAPDH antibodies were from Cell Signaling Technology. IL-6 and IL-10 ELISA kits were bought from R&D systems (nochnebiotech brand).

***Cell culture***

The rat intestinal epithelial cell line IEC-6 was bought from American Type Culture Collection (ATCC) and maintained in DMEM supplemented with 4 mM L-glutamine, 0.1 Unit/ml bovine insulin, 10 % fetal bovine serum, 1% penicillin/streptomycin at 37 °C in a humidified 5% CO_2_ incubator.

***Cell proliferation measurements by MTT***

5×10^3^ IEC-6 cells were seeded into a 96-well plate in 100 ml DMEM supplemented with varying concentrations (0, 12.5, 25, 50, 100, 200, 400 µM) of salidroside. For each concentration, five repeated wells were prepared (n=5) and a blank control group with culture medium only was also set, and then they were cultured 24 hr or 48 hr, respectively. After that, the cell viability was measured with an MTT kit following the manufacturer’s instructions. Briefly, the medium was removed and replaced by 100 µl of fresh phenol red-free culture medium. 10 μl MTT reagent was gently loaded into each well, and then the cells were cultured at 37 ^°^C foourshr. Seventy-five µl of the medium was removed from each well and then 50 µl DMSO was added into each well and mixed thoroughly w thea pipette. The 96-well plate was then incubated at 37 ^°^C forutesmin. Then the samples were mixed again and the optical density (OD) was measured at 540 nm for each well by a plate reader (EON, BioTek, USA).

**Cytokines production measurement by ELISA**

1×10^4^ IEC-6 cells were seeded in a 48-well plate in 0.5 ml fresh DMEM medium and were cultured for 24 hr. Then they were treated with different concentrations of salidroside (0, 12.5, 25, 50 µM) fo 2 hr ahead of stimulation by 0.1 mg/ml LPS. For each concentration, three repeated wells were prepared (n=3). The concentration of IL-6 and IL-10 in the supernatant was measured usng aan ELISA kit according to the manufacturer’s instructions. Briefly, the ELISA plate was firstly coated with the capture antibody, after washing, the plate was blocked with a diluent reagent, and then the supernatant samples or standards were loaded, incubating foourshr at room temperature. After washing, detection antibody was added, followed bourshr of incubation at room temperaturateThe substrate solution was loaded after washing and the plate was incubated for another 20 min at room temperature. After washing, stop solution was added and the plate was immediately subjected to a microplate reader (EON, BioTek) for optical density measurements at 450 nm.

***RNA isolation and quantitative reverse transcription PCR***

After LPS stimulation, the IEC-6 cells (for each concentration of salidroside, three repeated wells were prepared, n = 3) were pooled and lysed by TRIzol. Then the total RNA was extracted using the phenol-chloroform-isopropanol method. The first strand cDNA rsedreverse transcribed with Oligo (dT)15 (TaKaRa) and the extracted RNA samples, which was used for subsequent qRT-PCR with SYBR® Green Master Mix. The gene-specific primers used in qRT-PCR were as follows (5’–3’): *Il-6* (forward: ACTCACCTCTTCAGAACGAATTG; reverse: CCATCTTTGGAAGGTTCAGGTTG); *Il-10* (forward:

GACTTTAAGGGTTACCTGGGTTG; reverse: TCACATGCGCC

TTGATGTCTG); *Defa5* forward: AGACAACCA (GGACCTTGCTAT; 

reverse: GGAGAGGGACTCACGGGTAG); *Defa6* (forward: CTGAGCCACTCCAAGCTGAG; reverse: GTTGAGCCCAAAGC

TCTAAGAC). The experiment was performed on a Bio-Rad real-time PCR machine CFX6.

***Protein extraction and Western Blotblot analysis***

After LPS treatment, the IEC-6 cells (for each concentration of salidroside, three repeated wells were prepared, n=3) were pooled and washed with PBS twice, then lysed by RIPA buffer. The same volume of cell lysates was mixed with 4 × reducing loading buffer and then these samples were boiled for 10 min. After that, the total protein concentrations of all samples were quantified with a BCA kit. Then equal amounts of proteins were subjected for SDS-PAGE electrophoresis, the separated proteins in the gel were transferred to a PVDF membrane, which was subsequently blocked by 10% BSA and incubated with the indicated primary antibodies for the target proteins. After TBST washing for three times, the membrane was then incubated with the corresponding HRP-conjugated secondary antibodies. After TBST washing, the PVDF membrane was then incubated with ECL substrate and used for film exposureFor the darkroom. The phosphorylated proteins and the corresponding total protethey are measured in two different gels. The phosphorylation levels of target proteins in different signaling pathways were normalized to their corresponding total proteins, respectively. β-actin and GAPDH were used as the loading controls. 

***Data analysis***

All experiments were performed at lefor three times, data were expressed as the mean±standard deviation (SD). Statistical analyses were performed using GraphPad Prism 6 (GraphPad Software, Inc.). Statistical significance was determined as indicated in the figure legent. One-way analysis of variance (ANOVA) followedukeyTukey’s *post hoc* test was used to test for multiisoncomparisons.

## Results

***Salidroside suppressed the proliferation of intestinal epithelial cells***

To investigate the direct effect of salidroside on intestinal epithelial cells, we firstly cultud ofrat intestinal epithelial cell lat, IEC-6, with different concentrations of salidroside for 24 or 48 hr. Then, we applied an MTT kit to measure the cell viabilminefor examining the influence of salidroside on the proliferation and survival of intestinal epithelial cells. As expected, salidroside could suppress the proliferation of IEC-6 cells and showed evident cytotoxicity with higher concentrations. Specifically, the cell viability was getting decreased with the dose increment of salidroside (*P*<0.05), and longer time of treatment could further dampen the cell viabilip (*P*<0.05) (Figures 1A-B), which showed an obvious dose and time-dependent feature. Based on this result, we chose three lower doses of salidroside, 12.5, 25, and 50 µM, which were represented as low, medium and high concentrations of salidroside, respectively, to use in the following studies.

***Salidroside inhibited the up-regulation of IL-6 induced by LPS***

To study the effect of salidroside on cytokine secretion of intestinal epithelial cells under pathological circumstances, we firstly treated IEC-6 cells with different concentrations of salidroside foourshr ahead of LPS triggering. Then, LPS was applied to stimulate the intestinal epithelial cells to mimic the inflammatrredoccurring in enteritis and IBD. One typical pro-inflammatory cytokine-L-6, and one typical anti-inflammatory cytokine-I-10, were determined by ELISA. After LPS triggering, the production of IL-6 dramatically increasp (*P*<0.001), whereas salidroside was able to partially suppress the secretion of Iwithin a dose-dependent manner (for 25 µM, *P*<0.05; for 50 µM, *P*<0.01) (Figure 2A). On the contrary, IL-10 production was not so sensitive to LPS triggering and salidroside pre-treatment, though it had a decreasing trend without statistical differen (Figure 2B). In addition, the IL-6/IL-10 ratio was significantly increased after LPS treatmep (*P*<0.01) and showed no evident differences with salidroside treatme (Figure 2C). Here, we proved that salidroside could inhibit tionup-regulation of IL-6 induced by LPS. However, it had a negligible effect on IL-10 production.

***Salidroside suppressed the transcription of IL-6***

To confirm the above ELISA result, we further studied the effect of salidroside on cytokine secretion of intestinal epithelial cells on the transcriptional level. IEC-6 was treated without or with low (12.5 µM), medium (25 µM) and high (50 µM) concentrations of salidroside fo 2 hr, and then further incubated with LPS to induce the production of IL-6 and IL-10, which was measured by quantitative PCR (qPCR). Consistent with the ELISA results, the qPCR results also showed that salidroside could suppress tionup-regulation of IL-6 on mRNA level with an obvious dose-dependent feature (for 25 µM, *P*<0.05; for 50 µM, *P*<0.01) (Figure 3A). In contrast, the transcription of IL-10 showed no difference (*P*>0.05) after salidroside treatme (Figure 3B). Moreover, the IL-6/IL-10 ratio on transcriptional level was significantly decreased by salidroside (for 25 µM, *P*<0.05; for 50 µM, *P*<0.01) (Figure 3C). Collectively, we have confirmed that salidroside could suppress the expression of IL-6 on both transcriptional and translational levels, whereas it had no significant effect on IL-10.

***Salidroside suppressed the activation of NF-κB, MAPK, and STAT3 signaling pathways***

To explore the underlying mechanism of salidroside’s effect on intestinal epithelial cells, we checked some canonical signaling pathways involved in the IBD, such as NF-κB, MAPK, and JAK-STAT, throternWestern blotting. IEC-6 cells were treated without or with low (12.5 µM), medium (2 µM), and high (50 µM) concentrations of salidroside fo 2 hr, and then further treated with LPS to induce the activation of NF-κB, MAPK, and JAK-STAT signaling pathways, which were indicated by the increased phosphorylation levels of key proteins that were measuredternWestern blottip (*P*<0.05) (Figures 4A-B). Consistently, salidroside treatment could indeed suppress the phosphorylation of NF-κB, JNK, P38, JAK2, and STAp (*P*<0.0.5) (Figure 4C). However, the phosphorylation of Erk1/2 was not significantly impairp (*P<*0.05). The results here clearly demonstrated that salidroside could attenuate the cytokine production of intestinal epithelial cells induced by LPS through dampening NF-κB, MAPK, and STAT3 signaling pathways.

***Salidroside rescued the secretion of defensins in intestinal epithelial cells under inflammatory circumstances***

To verify whether salidroside can affect the expression of human defensins (HD) in intestinal epithelial cells under pathological conditions, we pre-incubated IEC-6 cells with low (12.5 µM), medium (2 µM), and high (50 µM) concentrations of salidroside foourshr, and then further treated cells with LPS or SAC for stimulation. The transcription of human defensin alpha 5 (HD-5) and alpha 6 (HD-6) was severely down-regulated after LPS (*P*<0.01) or SAC (*P*<0.001) treatment. However, salidroside pre-incubation could partially recover their expressip (*P*<0.05) (Figures 5A-B), which would help to clear the invading pathogens and suppress the inflammation of IEC-6 cells. This finding uncovered a novel salidroside mechanism side could suppress the progression of IBD through maintaining the expressions of HD-5 and HD-6 in intestinal epithelial cells, which could keep the integrity of physical barriers of theutto against the external pathogens. We speculated that this mechanism might apply to other inflammatory diseases treated by salidroside, such as asthma and endotoxemia.

## Discussion

In this study,stlyfirst checked the effects of salidroside on intestinal epithelial cells, the result showed that salidroside could directly inhibit the survival and proliferation of intestinal epithelial cewithin a dose and time-dependent manner. The cytotoxicowedshown here may depend on the cell type, for there is one report showing that even high concentration of salidroside had no obvious cytotoxicity on RAW264.7 macrophage cells (10). Meanwhile, this side-effect may harm the potential of salidroside to relieve IBD, although it cannot reach so high a concentration* in vivo*. Nevertheless, we chose three low doses of salidroside to perform our experiments, which showed relatively lower cytotoxicity. To determine the anti-inflammatory effect of salidroside, we measured the productions of IL-6, one of the representative pro-inflammatory cytokines, and IL-10, one of the representative anti-inflammatory cytokines, on both transcriptional and translational levels with intestinal epithelial cells stimulated by LPS. Consistent with previous publications (10, 30-32), we also observed the decreased production of IL-6. The down-regulated IL-6 and IL-6/IL-10 ratio confirmed the anti-inflammatory effect of salidroside for enteritis and other inflammatory bowel diseases. 

To reveal the underlying mechanism for the anti-inflammation function of salidroside on intestinal epithelial cells, we further examined the protein contents of NF-κB, MAPK, and JAK-STAT3 signaling pathways that were involved in the inflammation process. Extensive evidence had shown that salidroside could suppress the activation of these three pathways in the inflammation-mediated endotoxemia (10), ultraviolet-induced skin inflammation (30), D-galactose-induced Alzheimer’s disease model (31), and acute lung injury mouse model (32), respectiely. Consistently, our result also supported this conclusion. The phosphorylation levels of representative proteins in NF-κB, MAPK, and JAK-STAT3 signaling patysgot declined with the increasing dosages of salidroside, which further confirmed that salidroside inhibited the inflammation through attenuating NF-κB, MAPK, and JAK-STAT3 signaling pathways. Another exciting finding was the rescued expression of defensins by salidroside in intestinal epithelial cells, although the expression difference between SAC and SAC plus salidroside treated cells was not statistically significant. HD-5 and HD-6 were specifically expresseenethPenneth cells of the small intestine, ongedbelonging to antimicrobial and cytotoxic peptides involved in host defense and microbial balance regulation. This finding indicated that salidroside had multiple ways to suppress the inflammation, not only limite the cytokine.

## Conclusion

In general, our study revealed that salidroside could suppress the secretion of pro-inflammakinescytokine IL-6 through attenuating the NF-κB, MAPK, and JAK-STAT3 signaling pathways. At the same time, salidroside reinstated the production of human defensin 5 and 6 in intestinal epithelial cells after LPS-induced inflammation. All the evidence here demonstrated that salidroside possessed the potential to relieve IBD, which could become one appealing target for drug development in the future.
